# Prospective Evaluation of the Efficacy of Isolated Balloon Eustachian Tuboplasty: Short- and Medium-term Follow-up Results Based on Tubomanometry, ETDQ-7, Tympanometry, and Valsalva Maneuver

**DOI:** 10.1055/s-0043-1767804

**Published:** 2023-09-19

**Authors:** Cátia Azevedo, Filipa Moreira, António Fontes Lima, Fernando Milhazes Mar, Sérgio Vilarinho, Luís Dias

**Affiliations:** 1 Otorhinolaryngology Service, Hospital de Braga, Braga, Portugal

**Keywords:** eustachian tube, acoustic impedance tests, eustachian tube surgery

## Abstract

**Introduction** Balloon eustachian tuboplasty (BET) allows the treatment of the main etiology of eustachian tube disfunction (ETD).

**Objective** To evaluate the efficacy of isolated BET, through objective and subjective results, in the short and medium term, in patients with chronic obstructive ETD.

**Methods** Adult patients diagnosed with chronic obstructive ETD who underwent BET between January 2018 and December 2020 were enrolled in the study. We performed a prospective observational study of BET efficacy, by comparing subjective data, based on the Eustachian Tube Dysfunction Questionnaire-7 (ETDQ-7), and objective data, obtained by tympanometry, objective Valsalva maneuver and tubomanometry, prior to BET with these outcome tools on postprocedure follow-up.

**Results** In total, 30 BETs were performed and analyzed. There were no complications with the procedure. Analysis of BET efficacy was performed in the short-term (average of 7.5 weeks) and in the medium-term (average of 8 months). There was a significant reduction ( *p* < 0.0001) in the total ETDQ-7 score from baseline to both follow-up periods. A normalization of the ETDQ-7 score was observed in 60 and 83.3% of the performed procedures, in the short- and medium-term, respectively. In tubomanometry, we verified a significant improvement ( *p* < 0.0001) at all pressures, with a normalization of tubomanometry values in 53.3% and 43.3% of cases in the short- and medium-term, respectively. Tympanogram normalization occurred in 71.4% of patients with abnormal preoperative assessments.

**Conclusion** As an isolated procedure, BET results in significant improvements in symptomatology and objective metric results. This, associated with its safety profile, currently makes BET the most indicated therapeutic option in refractory obstructive ETD.

## Introduction

Eustachian tube dysfunction (ETD) is, since 2014, after an international meeting in Amsterdam with scientists and physicians with expertise in the field of Eustachian tube disorders, defined as a syndrome with a constellation of signs and symptoms suggestive of middle ear ventilatory dysfunction. 1 So, it is characterized by the recurrent otologic discomfort due to the persistent sensation of aural fullness, “under water” sensation, tinnitus, autophony, and/or muffled hearing. 2 Additionally, it may contribute to the onset or persistence of otitis media with effusion (OME), recurrent acute otitis media (AOM), atelectasic chronic otitis media (COM), and/or cholesteatomatous COM. 3 The consensus statement published by Schilder et al. 1 states that ETD may be divided into three types: dilatory or obstructive ETD, baro-challenge-induced ETD, and patulous ETD. In most cases, it results from a functional or anatomical obstructive dysfunction (that is, insufficient opening of the ET), which can be persistent or intermittent, and has an estimated prevalence of between 1 and 5% among adults. 4 Dilation of the cartilaginous portion of the ET with a balloon catheter, through balloon eustachian tuboplasty (BET), allows the treatment of the main etiology of ETD, leading to a reduction in intraluminal mucosal inflammation (longitudinal and circumferential crushing of inflamed mucosa and submucosa, and restoration of the ciliated pseudocolumnar epithelium) and improvement in ventilation and drainage of the middle ear. 5 In rare cases it may be associated with complications such as the creation of a false passage, AOM, patent Eustachian tube, tympanic membrane (TM) perforation, tinnitus, emphysema and hemotympanum. 6 Even so, it is a less invasive procedure when compared with other ET plasty techniques. Despite the promising results in the literature, the scientific evidence to determine the procedural effectiveness is still controversial, since the methods used for its evaluation are very variable, causing disagreement in the results and making their comparison difficult. Additionally, objective results based on tubomanometry (TMM), which is the most accurate assessment method, are still scarce.

Tubomanometry is a noninvasive, semiobjective, and quick test to evaluate ET function, regardless of the integrity of the TM. It assesses the necessary pressure threshold for ET opening and defines the opening latency (time delay between applying pressure to the nasopharynx and measuring pressure changes in the ear canal after opening of the ET). To perform TMM, defined pressures (30, 40 and 50 mBar) are transmitted to the nasopharynx through a nasal connection. During this transmission, the patient is asked to swallow. Swallowing will temporarily seal the nasopharynx and if the applied pressure in the nasopharynx is sufficient for ET opening, this same pressure will be transmitted to the middle ear. A pressure probe in the external auditory canal (EAC) will register pressure changes in the middle ear through movements of the tympanic membrane. Two pressure variation curves are displayed on the TMM monitor, one related to the nasopharynx and one to the EAC, and with these two curves the opening latency index (the so-called *R-value* ) is determined for each pressure. 7

The main goals of the present study were to determine the efficacy of BET, as an isolated procedure, in patients with chronic ETD (defined by the European consensus statement as an ETD that persists for ≥ 3 months 1 ) based on results obtained by TMM, the Eustachian Tube Dysfunction Questionnaire-7 (ETDQ-7), tympanometry, and Valsalva maneuver in a short- and medium-term follow-up and analyze the correlations between objective and subjective results.

## Materials and Methods

### Study Population

We performed a prospective observational study of adult patients (>18 years old) who underwent an isolated BET between January 2018 and December 2020. The included patients had evidence of obstructive ETD for > 6 months, confirmed by ETDQ-7 (cutoff > 14.5) and TMM (R >1 or nonmeasurable R, at least in 1 of the pressures), who have been refractory to conservative and medical treatment. The included patients had no history of chronic middle ear disease or risk factors for ETD such as marked weight loss, radiotherapy, or history of nasopharyngeal surgery. All patients had a clinical assessment which included a complete ENT examination with flexible nasopharyngoscopy, otoscopy, objective modified Valsalva maneuver, tympanometry, and pure tone audiometry, and underwent a preoperative temporal bone computed tomography (CT) scan to confirm the integrity of the internal carotid artery bone channel.

Exclusion criteria were obstruction of the ET opening in the nasopharynx due to adenoid tissue, patulous ET (TMM with R = 0 or near to 0, at all 3 pressures), perforated TM, cholesteatoma, patients with transtympanic ventilation grommet tubes, severe *pars tensa* atelectasis (Sade grade 4), obstructive deviated septum, uncontrolled chronic rhinosinusitis, previous ET operation or radiation treatment, syndrome known to be associated with Eustachian tube dysfunction or cleft palate, immunological deficiency, and dehiscent or aberrant carotid artery.

The present study was approved by the local ethic committee (Reference 206/2019) and all patients gave their informed consent prior to their inclusion in the present study.

### Procedure

At our institution, the BET procedure is performed transnasally, under general anesthesia. After positioning the patient in the supine position, with the head elevated, the ipsilateral nasal cavity is decongested (or bilateral if the procedure is performed bilaterally) with topical application of nasal decongestant. The procedure is performed under endoscopic visualization, usually using a 30° and 4-mm diameter endoscope. For BET, we use a single-use dilatation balloon (TubaVent, Spiggle & Theiss, Overath, Germany) with a size of 3.0 × 20mm. The balloon is introduced, through an insertion instrument, into the nasopharyngeal ostium of the ET and advanced into the cartilaginous portion of the ET. After fully inserted, the balloon is dilated with saline solution to a target pressure of 10 bar. This pressure is applied for 2 minutes, before being deflated and the device removed under endoscopic observation. In cases of bilateral dysfunction, the same procedure is repeated on the other side during the same operative time, using a new balloon for this purpose. The patients included in the present study had no other associated complementary procedure.

### Outcome Evaluation

The evaluation of BET efficacy was performed by comparing subjective data, based on the ETDQ-7, and objective data, obtained by tympanometry, objective TM mobility with Valsalva maneuver and TMM, prior to BET with these outcome tools on the postprocedure follow-up, performed in an outpatient setting.

The ETDQ-7 is a patient-report questionnaire used to quantify the ETD-related symptoms (pressure, pain, “under water” feeling, ear symptoms when presenting with cold/sinusitis problems, crackling/popping, ringing, and muffled hearing): a higher score is indicative of greater dysfunction. In bilateral cases, patients completed separate pre- and postprocedure side-specific ETDQ-7 for each ear. We used the validated ETDQ-7 to European Portuguese 8 and determined the total scale score cutoff value of 14.5 to define patients with or without ETD (ETDQ-7 maximum score: 49 points)

Improvement in Valsalva maneuver was defined as a change from negative before BET to positive Valsalva at follow-up; tympanometry normalization was defined per side as a change from type B or C at baseline to type A at follow-up.

For TMM, the opening latency index (R value) was determined for each applied pressure. This index, calculated through the equation R = P1–C1/C2–C1, is a measure of the time delay between applying pressure to the nasopharynx and measuring pressure changes in the ear canal after opening of the ET. “P1” is identified in the pressure variation curve of the EAC, which represents the beginning of the TM movement or the increase in pressure in the EAC after the application of pressure in the nasopharynx. “C1” and “C2” are determined on the nasopharyngeal pressure variation curve and represent the beginning of nasopharyngeal pressure rise and the maximum peak pressure reached in the nasopharynx, respectively. 9 The latency quantifies ET function since R < 1 indicates normal ET function with almost immediate opening, R ≥ 1 indicates a delayed opening of the ET, nonmeasurable R indicates a complete obstruction of the ET, and R = 0 or fluctuation on the pressure variation curve of the ear canal indicates a patulous ET. R values cutoff points were defined by Estève et al. in 2001 10 and they verified that this parameter is not influenced by the different pressure intensities applied. For this study, TMM normalization was defined per side as a change from R > 1 or nonmeasurable R in at least one of the evaluated pressures before BET (that indicates a delay or absence of ET opening, respectively) to R < 1 at all pressures (30, 40 and 50 mBar) after BET.

### Statistical Analysis

Categorical variables were summarized by frequency (N) and percentage. Continuous variables were described as median and interquartile range (IQR). Treatment outcomes were evaluated in two different evaluation moments for each patient (shot- and medium-term follow-up after BET) and compared with the baseline results using the Wilcoxon signed-rank test and the Friedman test for continuous variables, and the McNemar and Cochrane Q test for dichotomous measures. The Mann-Whitney U test was used to compare differences in ETDQ-7 scores between two independent groups. Data analysis was performed using IBM SPSS Statistics for Windows version 26 (IBM Corp., Armonk, NY, USA), and p-values < 0.05 were considered significant.

## Results

In the present study, 30 BETs were performed and analyzed (14 right-sided and 16 left-sided, in a total of 18 patients) in ears with an isolated diagnosis of chronic obstructive ETD. All patients had a preoperative ETDQ-7 score > 14.5 and TMM with opening latency index (R value) > 1 or nonmeasurable, at least in 1 of the pressures. Regarding tympanometry, 14 patients (46.7%) had a type C or type B tympanogram prior to BET. R values obtained by TMM and the respective types of tympanograms are summarized in Table 1 : as the severity of ET dysfunction increases (more severe if dysfunction at high pressures), the proportion of patients with type A tympanogram decreases. The cohort included 7 men (38.9%) and 11 women (61.1%); the average age was 47.2 years old (19 to 69 years old). All patients were taking local nasal corticosteroids and had performed nasal irrigation for at least 3 months, with no effect, prior to being included in the study. Analysis of BET efficacy was performed in the short-term (after an average of 7.5 weeks) and in the medium-term (after an average of 8 months). Balloon eustachian tuboplasty was overall well tolerated an in all the procedures performed, there were no intra- and/or postoperative complications.

**Table 1:** Preoperative tympanometry results according to the R values obtained by tubomanometry for each evaluated pressure

	Tympanometry		
TMM measurement	Tympanogram Type A *n* (%)	Tympanogram Type C *n* (%)	Tympanogram Type B *n* (%)
R > 1 or nonmeasurable R – 30 mBar ( *n* = 30)	16 (53.3)	8 (26.7)	6 (20)
R >1 or nonmeasurable R – 40 mBar ( *n* = 28)	14 (50)	8 (28.6)	6 (21.4)
R > 1 or nonmeasurable R – 50 mBar ( *n* = 24)	10 (41.7)	8 (33.3)	6 (25)

Abbreviations: TMM, Tubomanometry; R - opening latency index of Eustachian Tube.

### Eustachian Tube Dysfunction Questionnaire-7 (ETDQ-7)

All patients showed an immediate improvement in the ETDQ-7 score after BET compared to baseline. During follow-up, patient-reported complaints continued to improve in 21 (70%) patients and remained stable in 7 (23.3%). In 2 patients, despite the initial improvement, there was a further deterioration of the subjective complaints in the medium-term evaluation.

**Fig. 1:**
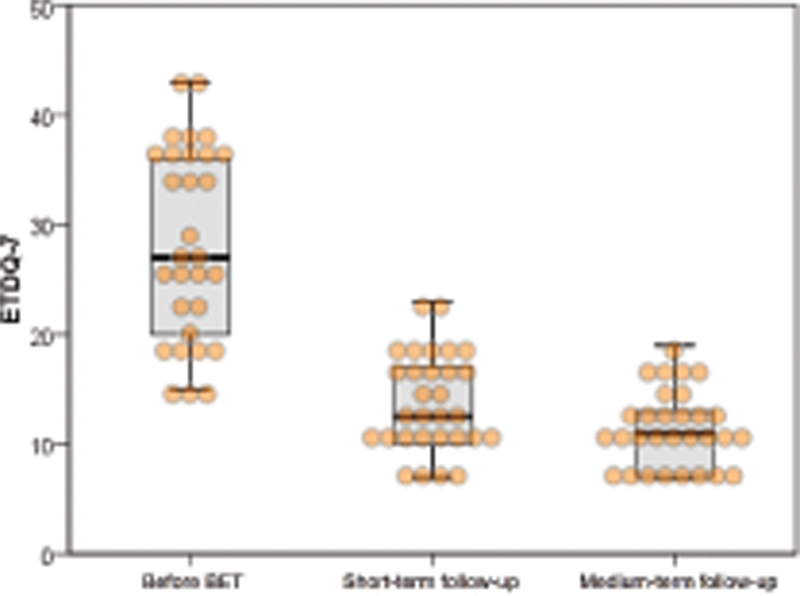
ETDQ-7 results. Higher scores signify greater dysfunction. Values are presented as medians.

Overall, there was a statistically significant reduction (p < 0.0001) in the total ETDQ-7 score from baseline to both follow-up periods (Baseline: median 27, IQR 16.5; Short-term follow-up: median 12.5, IQR 7.25; Medium-term follow-up: median 11, IQR 6.25) ( Fig. 1 ). A normalization of the score of the ETDQ-7 (< 14.5) was observed in 60 and 83.3% of the performed procedures, in the short- and medium-term, respectively.

### Tubomanometry (TMM)

Matched pre- and post-BET results showed an immediate improvement with TMM normalization in all pressures in 16 (53.3%) patients, but only in 13 patients was this improvement sustained over the follow-up period, with 5 patients showing a medium-term deterioration at 30 and 40 mBar (R > 1 or nonmeasurable R); in 2 patients this improvement was only seen in the medium-term follow-up.

Four (13.3%) patients only showed an improvement on TMM at 40 and 50 mBar and 6 (20%) patients only at 50 mBar. Two (6.7%) patients showed no improvement on TMM at any pressure.

Overall, there was a statistically significant improvement (p < 0.0001, Cochrane Q test) in the TMM results in the 2 postoperative periods at all pressures (30, 40 and 50 mbar), compared with the baseline results, with a normalization of TMM values (R < 1 at all pressures) in 53.3 and 43.3% of cases in the short- and medium-term, respectively ( Table 2 ). No patulous ETD was recorded after the procedure (R = 0 on TMM at all three pressures). There were no statistically significant differences between the short- and medium-term follow-up results on TMM.

**Table 2:** Tubomanometry results

	Before BET *n* (%)	Short-term FU *n* (%)	Medium-term FU *n* (%)
Obstructive ETD (R >1 or nonmeasurable R in at least one of the evaluated pressures)	30 (100)	14 (46.7)**	17 (56.7)**
R < 1–30 mbar	0 (0)	16 (53.3)**	13 (43.3)**
R < 1–40 mbar	2 (6.7)	19 (63.3)**	17 (56.7)**
R < 1–50 mbar	6 (20)	26 (86.7)**	27 (90)**

Abbreviations: R - opening latency index of Eustachian Tube; BET, Balloon Eustachian Tuboplasty; ETD, Eustachian Tube Dysfunction; FU, Follow-up.

** *p* < 0.001, compared with baseline (preoperative) results. Cochrane Q test.

### Tympanometry and Valsalva Maneuver

**Fig. 2:**
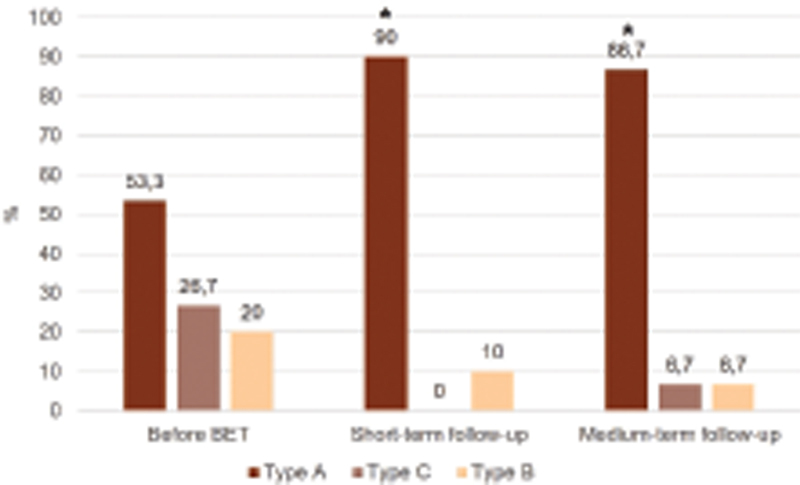
Tympanogram results. *p < 0.05 versus before BET.

In our study population, tympanogram normalization (type A) occurred in 10 (71.4%) out of 14 patients with abnormal preoperative assessments. Overall, there was a statistically significant increase ( *p* < 0.05) in type A tympanogram number during the follow-up period after BET ( Fig. 2 ). There was one case with short-term normalization of the tympanogram, with recurrence of the dysfunction in the medium-term evaluation.

Regarding the ability to perform the Valsalva maneuver, the proportion of those able to successfully perform it had a statistically significant (p < 0.05) increase from 53.3% at baseline to 76.6% at the short-term time point and to 93.3% at the medium-term follow-up. The 16.7% increase from the 2 postoperative time points were not statistically significant ( *p* = 0.231).

### Correlations Between Objective and Subjective Results

In the medium term, we found that patients with objective ETD on TMM and tympanogram tend to have higher ETDQ-7 scores compared with patients without objective ETD (median ETDQ-7 score = 13 in patients with ETD versus median ETDQ-7 score = 10 in patients without ETD), although these differences were not statistically significant ( *p* > 0.05).

## Discussion

The currently available treatments for ETD, although various, do not have a clear proven efficacy and have some essential limitations: medical treatment ends up being generalized and not directed to the underlying etiology of ETD (like nasal topical decongestants or steroid sprays); placement of transtympanic ventilation tubes have only a temporary effect, as it does not reduce the inflammation and the inherent pathology of ET; surgical techniques that aimed to widening the ET opening (like LASER or microdebrider) have no effect on the inflamed tissue within the lumen of the ET. 4 11 12 Thus, BET emerged to overcome these previously recognized weaknesses, having been performed for the first time in Europe in 2010 by Ockermann et al. 13

Regarding its safety profile, there are just few reported adverse effects. In a study carried out by Anand et al., 4 in which 235 BET procedures were performed, there were two occurrences of patulous ET, one of which resolved spontaneously, and a false passage occurred in one patient. The authors consider that patulous ET occurred mainly in patients who did not have a demonstrable burden of inflammatory disease within the opening of the ET on preoperative examination. Schroder et al. 14 described only the occurrence of 3 in > 1,000 procedures performed of emphysema within the parotid region, that reabsorbed without sequelae and without permanent damage of the ET. In our study, no procedure-related adverse events were reported, which is in agreement with other studies, 11 but it can also be due to the low number of procedures performed

Regarding the effectiveness of BET in the treatment of ETD, several studies and systematic reviews on the subject have been published in the past decade since its introduction into clinical practice. 3 6 15 16 17 18 19 To evaluate BET results, multiple tools, both subjective and objective, have been used that assess ET function. Tympanometry remains the most frequently used test in most studies to objectively report ET function. Fontes Lima et al. 20 showed that despite having a good specificity (92.6%) for the detection of ETD, its sensitivity is quite limited (61.1%) and therefore it is unsuitable and insufficient for excluding obstructive ETD, especially in patients with suspicious symptoms. Additionally, this test is also not appropriate for diagnosing intermittent ETD since middle ear pressure is highly variable throughout the day in these cases. Thus, tubomanometry emerged as a tool with greater accuracy that also allows a quantitative assessment of ET function. Its high sensitivity (91.7% 20 ) allows the diagnosis of milder ETDs, especially in patients with classic symptoms but a normal TM exam and a type A tympanogram. Fontes Lima et al. 20 concluded that when used alone, tympanometry is insufficient, ETDQ-7 may overestimate, and TMM is the most accurate of the three. In this context, as none of them is completely reliable and representative of the real function of the ET, they are often used together in a complementary way to increase diagnostic sensitivity and specificity. Considering the subjective assessment measures, we used the ETDQ-7 score, which is practically universally used to report patients complaints related to ETD. In our study, a total ETDQ-7 score < 14.5 (considered normal) was observed in 60% of procedures in the short-term evaluation and 83.3% in the medium-term follow-up. All patients showed an immediately improvement in the ETDQ-7 score after BET compared with baseline. Only in 2 patients, despite the initial improvement, there was a further deterioration of the subjective complaints in the medium-term evaluation. These results are in agreement with multiple studies already published in the literature, which evaluated BET as an isolated treatment for ETD: Anand et al. 4 reported a normalization in ETDQ-7 in 55.5% of patients (79 of 142) at 6 weeks of follow-up, referring that in the long-term follow-up (at 52 weeks) a significant degree of durability of these results was demonstrated, with normalization of the score in almost 40% of the study population; in the systematic review and meta-analysis performed by Froehlich et al., 3 which included 12 studies and 448 patients who only underwent BET, revealed a statistically significant improvement in subjective measures, with normalization of the ETDQ-7 scores in 53.3% of patients in the short-term follow-up and in 58.9% in the long-term (12 months) follow-up.

The American Academy of Otolaryngology–Head and Neck Surgery (AAO-HNS) published a Clinical Consensus Statement where they refer that the subjective assessment performed with the ETDQ-7 is insufficient to establish a diagnosis of obstructive ETD 21 since it can be influenced acutely by infectious or non-ETD-related factors, and so the evaluation with objective measures of the ET function is essential. For the objective evaluation of the ET function, we used tympanometry, the ability to perform the Valsalva maneuver, and TMM. In our study population, tympanogram normalization (type A) occurred in 71.4% (10 of 14) patients with abnormal preoperative assessments. There was one case with short-term normalization of the tympanogram, with recurrence of the dysfunction (type C) in the medium-term evaluation. These results are similar to those reported by Cutler et al. 22 but slightly better than those found in the rest of the literature: Anand et al. 4 reported a normalization of 57.4% at 6 weeks and 39.6% at 52 weeks; Froehlich et al. 3 stated a tympanogram normalization in 45%, with stability in 50.5% of cases at the long-term follow-up; in a randomized controlled trial performed by Poe et al., 11 tympanogram normalization occurred in 62.2% in the BET group at 24 weeks postoperatively, which was significantly higher than the 8.5% in the control cohort (medical treatment alone). However, improvement (not normalization) results, which include normalization and type B baseline tympanograms that improved to type C cases, appear to be better. 3 4

There is also already a consensus regarding the value of the ability to perform the Valsalva maneuver as an outcome assessment after BET. 21 In our study, at baseline, 53.3% had the capacity to perform a Valsalva maneuver, which increased up to 76.6 and 93.3% at the short- and medium-term follow-up, respectively. A greater increase in the percentage of cases able to perform the Valsalva maneuver after BET was observed on a meta-analysis 3 where there was an increase from 13 to 71 and 81% in the short-term and long-term time points, respectively. Poe et al. 11 described a 32.8% increase in the number of ears with positive modified Valsalva maneuver in the investigational arm (BET group) versus 3.1% in the control group (medical management). Cutler et al. 22 reported an improving from 28.3% at baseline to 73.9% at the long-term follow-up (mean of 29 months).

Finally, the literature regarding the use of objective results obtained by TMM to assess BET efficacy in ETD is still quite scarce. In fact, and despite its accuracy, few physicians (only 6%) perform an objective assessment with TMM prior to BET, as demonstrated in surveys carried out by members from the American Rhinologic Society and American Otological Society. 23 Recently, TMM results were, combined with subjectively positive Valsalva maneuver and clicking sound when swallowing, included in the eustachian tube score (ETS), 7 facilitating quantification and interindividual comparison of ET function. It is recommended to measure ET function at 30, 40 and 50 mBar to determine the severity of ETD, as patients with mild obstructive dysfunction may have R values > 1 only at low pressures (30 mBar), with normal measurements (R < 1) at 40 or 50 mBar. A dysfunction of the ET opening at increased pressures (50 mBar) generally means a severe ETD, and, in these cases, an impairment of ET opening at lower pressures is also present. 9 We observed an overall statistically significant improvement in TMM results in the 2 postoperative periods at all pressures (30, 40 and 50 mbar), compared with the baseline results, with a normalization of TMM values (R < 1 at all pressures) in 53.3 and 43.3% of cases in the short- and medium-term, respectively. Schroder et al. performed various studies on this subject, 9 14 including the TMM as a measure to evaluate the results after BET, although integrated in the ETS. They found that there was a significant improvement in ETS in 71% of cases after 2 months of BET (506 dilatation procedures were included) and in 73% after 1 year (188 dilatation procedures were included). However, these results cannot be fully compared with ours as they do not exclusively assess the TMM results. Schmitt et al. 24 observed an improvement in 81% of matched pre- and postoperative TMM performed in 30 procedures, including 33% normalization, and concluded that TMM seems to be more reliable and less dependent on middle ear status than other objective measures. However, in this article, the evaluated study population was heterogeneous, with other procedures being performed in addition to BET, so the results cannot be fully extrapolated as being a direct result of BET. Only Xiong et al. 25 demonstrated a progressive improvement in the opening latency index in all pressures during the 1 ^st^ year of follow-up after BET (study carried out in 58 procedures).

In our study, we focused mainly on the normalization of the subjective and objective measures evaluated, which can reduce and even underestimate the real effectiveness of the procedure: a significant improvement in complaints and objective measures can occur in patients with severe pathology at baseline, even if normalization does not occur. Therefore, some articles prefer to focus on improvement instead of normalization of measures and this may also explain the absence of a statistically significant correlation between both forms of effect measures evaluated in our study, which is in agreement with the study of diagnostic accuracy performed by Fontes Lima et al., 20 who concluded that the correlation between the ETDQ-7 and tympanometry and between the ETDQ-7 and TMM were weak. The incomplete agreement between the normalization of the ETDQ-7 and TMM can also be explained by the fact that some patients with only dysfunction of the ET opening at lower pressures (30 mbar) on TMM (mild dysfunction) may be totally asymptomatic. However, the relationship between objective and subjective measures (tympanometry and ETDQ-7, respectively) has already been demonstrated (an improvement in tympanometry was associated with normalization of ETDQ-7 score), 11 although sometimes the proportion of patients with symptomatic improvement is greater than the proportion of improvement in functional outcomes assessed by tympanometry. 4 In the present study, given the aim of evaluating the effectiveness of BET as an isolated procedure, patients undergoing other adjuvant procedures were not included. However, we must remember that ETD is not an isolated pathology in most cases and inflammation that occurs inside the ET is often a consequence or associated with inflammation at other sites. Therefore, the association of BET with other procedures when clinically indicated, such as turbinate reduction, nasal or sinus surgery or adenoidectomy, can also increase the effectiveness of this procedure, which is especially important to prevent recurrence.

### Strengths, Weaknesses, and Future Research Directions

As strengths of the present study, we highlight the fact that it is a prospective study with a controlled postoperative follow-up, with the inclusion of a very homogeneous sample that was exclusively submitted to BET. Additionally, it is one of the few studies that evaluate the objective results obtained by TMM, which is more sensitive and more reliable than tympanometry. Despite these aspects, it is a study with a very limited number of patients, and the long-term effectiveness of the procedure was not evaluated.

Future research, ideally randomized and blind controlled trials that evaluate the effectiveness of the procedure using the TMM as the tool for objective evaluation of the ET function, minimizing the risk of a placebo effect, is warranted to address additional controversies related to BET.

## Conclusions

Balloon eustachian tuboplasty, as an isolated procedure in obstructive ETD, results in significant improvements in symptomatology and objective metric results that appear to be stable over time. These promising results, associated with the good safety profile, currently make balloon tuboplasty the most indicated therapeutic option in the treatment of chronic obstructive ETD, whenever other comorbidities that may be associated with ETD are excluded and/or treated.
